# *De novo* and recurrent post-transplant membranous nephropathy cases show similar rates of concurrent antibody-mediated rejection

**DOI:** 10.3389/fneph.2024.1438065

**Published:** 2024-09-03

**Authors:** Nikka Khorsandi, Hwarang Stephen Han, Raja Rajalingam, Jun Shoji, Anatoly Urisman

**Affiliations:** ^1^ Department of Pathology, University of California, San Francisco, San Francisco, CA, United States; ^2^ Department of Medicine, Nephrology Division, University of California, San Francisco, San Francisco, CA, United States; ^3^ Department of Internal Medicine, Dell Medical School, University of Texas at Austin, Austin, TX, United States; ^4^ Immunogenetics and Transplantation Laboratory, Department of Surgery, University of California, San Francisco, San Francisco, CA, United States; ^5^ Department of Medicine, Transplant Nephrology, Cedars-Sinai Medical Center, Los Angeles, CA, United States

**Keywords:** membranous nephropathy (MN), transplant kidney, transplant kidney pathology, antibody mediated allograft rejection, *de novo* membranous nephropathy, recurrent membranous nephropathy

## Abstract

**Background:**

Membranous nephropathy (MN) can develop post-kidney transplant and is classified as a recurrent disease in patients with a history of MN in the native kidneys or as *de novo* disease in patients without such history. The mechanism of recurrent MN is thought to be like that of primary MN, but the mechanism of *de novo* MN is not well delineated. An association between *de novo* MN and antibody-mediated rejection (AMR) has been suggested.

**Methods:**

A search of the pathology database from our medical center identified 11 cases of recurrent and 15 cases of *de novo* MN, in which clinical and histologic findings were compared. No significant differences were identified in the demographic characteristics, serum creatinine and proteinuria trends, or rates of allograft failure between the recurrent and *de novo* MN groups.

**Results:**

Rates of concurrent AMR were high in both groups (36% and 40%, respectively) but not statistically different from each other. PLA2R immunofluorescence (IF) positivity was seen in 64% of recurrent MN cases compared to 33% of *de novo* MN cases, suggesting a higher incidence of PLA2R-positive *de novo* MN than previously reported. No significant histologic differences were identified in the initial biopsies from the two groups, except mean IgG intensity by IF was higher in the recurrent group, suggesting a higher load of immune complex deposits at diagnosis in this group.

**Conclusion:**

The findings do not provide support for a specific association between AMR and *de novo* MN, but whether there is a possible link between both forms of post-transplant MN and AMR remains an unanswered question.

## Highlights

- No significant clinical or histologic differences were identified among 26 recurrent and *de novo* membranous nephropathy (MN) patients.
- PLA2R positivity was identified in both cases of *de novo* MN (33%) and recurrent MN (64%).
- Similar rates of concurrent antibody mediated rejection (AMR) were seen in recurrent (36%) and *de novo* (40%) MN, suggesting a link between AMR and MN.

## 1 Introduction

**Figure 1:**
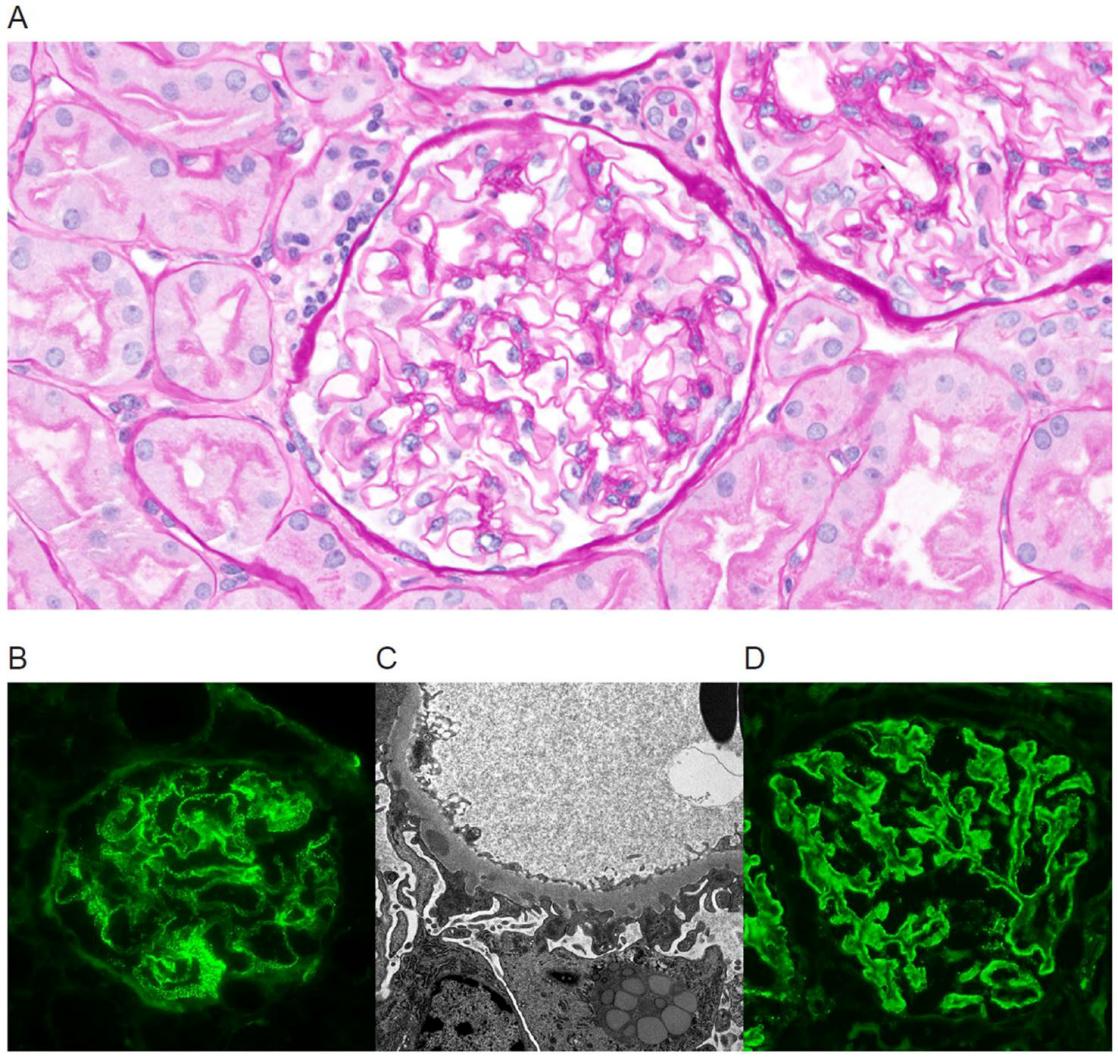
Kidney biopsy findings in a case of PLA2R-positive *de novo* MN in a 59-year-old patient with a history of ESRD from autosomal dominant polycystic kidney disease and no history of MN in the native kidney, who underwent a biopsy for proteinuria at 12 days post-transplant **(A–C)**. The initial and 6-month follow-up biopsies revealed no PLA2R staining in the glomeruli, but in a 12-month follow-up biopsy PLA2R stain was positive **(D)**. **(A)** Light microscopy at 12 months shows well-preserved renal parenchyma. The glomeruli have open capillary lumens with subtle thickening of the capillary loop walls. Proximal tubules contain increased cytoplasmic protein reabsorption droplets suggestive of proteinuria. **(B)** Immunofluorescence stain for IgG demonstrates granular staining along glomerular basement membranes. **(C)** Electron microscopy reveals scattered subepithelial electron-dense immune complex deposits with associated basement membrane remodeling and prominent podocyte foot process effacement. **(D)** Immunofluorescence PLA2R stain is positive at 12 months post-transplant.

Membranous nephropathy (MN) is an immune complex-mediated glomerular disease caused by the deposition of immune complexes along the epithelial surface of glomerular basement membranes (GBM) with associated podocyte injury and progressive GBM thickening and remodeling (1). The resulting damage leads to proteinuria and often progresses to renal failure (2). Immunofluorescence examination of the kidney biopsy tissue shows IgG and C3 granular staining along glomerular capillary walls, while electron microscopy demonstrates corresponding subepithelial electron-dense immune complex deposits, GBM remodeling, and effacement of the podocyte foot processes (**Figure 1**) (1, 3). MN is classified as either primary or secondary depending on the etiology of the glomerular injury. Primary MN was previously considered an idiopathic disease, but discoveries over the last decade point to an autoimmune response directed at specific podocyte antigens as the underlying mechanism. Autoantibodies directed against phospholipase A2 receptor (PLA2R) are found in 70-80% of primary MN cases (3, 4), and the list of less common target antigens is growing (5). Unlike primary MN, where no disease triggers are known, secondary MN represents approximately 20% of MN cases that develop in the setting of a well-defined association such as infection, drug exposure, malignancy, or systemic autoimmune diseases (e.g. systemic lupus erythematous) (6, 7). Autoantibody target discovery studies have shown that many antigens of primary MN are shared with those of secondary MN, consistent with a hypothesis that both primary and secondary MN have similar underlying autoimmune mechanisms (8, 9).

MN can develop in the allograft kidney after kidney transplant, and it is classified as “*de novo*” if it occurs in patients without a history of MN in their native kidneys, and “recurrent” if it occurs in patients with a history of MN (10). Recurrent MN has a reported incidence as high as 48% and is associated with increased allograft loss (11–13). In contrast, *de novo* MN is much rarer, with an incidence of around 2% (14). Recurrent MN is believed to result from the same underlying pathophysiology as primary MN, mediated by the continued presence of circulating autoantibodies after transplant. However, the exact mechanisms by which *de novo* MN develops are not fully understood, although several risk factors have been proposed, including hepatitis B or C infection and antibody-mediated rejection (7). PLA2R positivity in allograft biopsies of post-transplant MN is strongly associated with recurrent disease, with a reported rate of 83% in recurrent MN compared to only 8% in *de novo* MN (15).

It has been proposed that the target antigens in *de novo* MN may be different from those of recurrent MN (16). Specifically, a possible association between *de novo* MN and antibody-mediated rejection (AMR) has been suggested in several case reports, in which *de novo* MN biopsy findings coincided with histologic features of AMR and the detection of circulating donor-specific HLA antibodies (16, 17). To better understand the clinical and pathologic differences between *de novo* and recurrent MN, we retrospectively analyzed cases of biopsy-proven post-transplant MN at a single large renal transplant center.

## 2 Methods

The study protocol was approved by the University of California San Francisco Institutional Review Board (approval #20-31731). The institution’s clinical pathology database was searched for all renal allograft biopsies diagnosed with post-transplant MN between the years 2014 and 2020. Pathology reports were reviewed to confirm the diagnosis and to extract relevant histologic data including immunofluorescence staining intensity and pathologic features of antibody-mediated rejection.

Electronic medical records of each identified patient were then searched to extract the demographic and clinical data including the etiology of end-stage renal disease (ESRD) in native kidneys, serum creatinine, urine spot protein-to-creatinine ratio, HLA types of recipient and current donor, hepatitis B and C status, CMV status and long-term allograft outcomes. Data on serum anti-PLA2R antibody measurements was only available in three of the *de novo* cases and two of the recurrent cases and therefore was not included in the analysis.

Patients with post-transplant MN who had a history of MN in the native kidneys were categorized as having “recurrent” disease. Patients with no history of MN and a clear history of ESRD due to a non-MN disease in the native kidneys were categorized as having “*de novo*” disease. Patients with an unknown cause of ESRD in the native kidneys and patients with systemic lupus erythematosus were excluded from the study. Allograft failure was defined as the return to hemodialysis or re-transplant. One patient died from a cause unrelated to renal function and was censored from outcome analysis, more specifically analysis of allograft failure. In patients with multiple allograft biopsies, the patient’s MN was categorized as PLA2R positive if at least one biopsy demonstrated PLA2R IF positivity. Similarly, a patient was considered to have AMR if at least one biopsy met Banff diagnostic criteria for AMR. In all biopsies, histologic features of rejection and chronicity were recorded prospectively using the established Banff classification system (18, 19).

In patients with multiple biopsies, the first biopsy that established the diagnosis of MN was used as the representative, “diagnostic” biopsy when comparing the pathologic features in biopsies from different patients. The intensity of C4d, C3, and IgG immunofluorescence (IF) stains was recorded using a rating scale of 0-3. Kappa and Lambda IF intensity was classified as either being present or absent with presence defined as a score of at least 1 on the 0-3 scale. IgG subsets measured by IF were available only in two of the *de novo* cases and three of the recurrent cases and therefore were not included in the analysis.

HLA antibodies were measured in dithiothreitol-treated serum samples using the Luminex-based single antigen bead assay (One Lambda Inc., Canoga Park, CA) (20). Antibody specificity is determined based on known cross-reactivity patterns. The median fluorescence intensity (MFI) is used as an arbitrary unit of antibody quantity. If multiple beads have allelic variants of the same antigen (e.g., HLA-A*02:01, *02:03, *02:06—variants of HLA-A2 antigen), then the average MFI of all positive beads is used to quantify HLA-A2 antibody strength. LABXpress Pipettor (One Lambda), a high throughput liquid handling system to aspirate and dispense precise volumes into test wells of a 96-well reaction plate is used to minimize interassay variations. The donor-specific antibodies (DSA) were assessed by comparing them against the current donor’s HLA types. The DSAs and their MFI values were assessed in all sera samples collected at the time of any available pre-transplant points to either the point of allograft failure or to the most recent follow-up date available. Only DSA against the patient’s current transplant were included in the analysis. The DSA were classified as preformed (those detected prior to transplant) and *de novo* (those detected after the current transplant). *De novo* DSA (dnDSA) were further divided into those that were first detected prior to MN diagnosis or concurrent with the diagnosis. The MFI of each dnDSA was charted over time relative to the patient’s MN diagnosis. Concurrent urine spot protein to creatinine ratios (UPCR) were reviewed and co-plotted along MFI to evaluate for possible correlation between dnDSA levels and proteinuria levels.

Statistical analyses were conducted with R Statistical Software (v4.1.2; R Core Team 2021). Evaluation of differences between the *de novo* and recurrent groups was performed using Pearson’s chi-square tests or student’s unpaired t-test, when relevant. Prior to each t-test, a test to evaluate variance was performed and appropriately applied. Significance was defined as a probability of less than 0.05.

## 3 Results

Fifty-two biopsies with post-transplant MN were initially identified from 31 patients. After reviewing the electronic medical records, the etiology of ESRD was unclear in 5 patients, who were excluded from further analysis. Among the remaining 26 patients, 11 were classified as having recurrent MN and 15 as having *de novo* MN. The diagnosis of post-transplant MN was confirmed by electron microscopy in the representative biopsy for 19 patients, including 10 of 15 cases of *de novo* MN and 9 of 11 cases of recurrent MN. In the recurrent MN group, 3 biopsies were performed for an elevated creatinine level, 5 for proteinuria, 1 for both an elevated creatinine level and proteinuria, and 2 for routine surveillance. In the *de novo* MN group, 5 biopsies were performed for an elevated creatinine level, 4 for proteinuria, and 6 for routine surveillance.

We first compared the demographic and clinical variables of patients with recurrent and *de novo* MN and identified no significant differences between the two groups (**Table 1**). The average age of the patients in the recurrent MN group at the time of diagnostic biopsy was 51.7 years compared to 44.0 years in the *de novo* MN group. On average, a diagnosis of MN was made in the recurrent MN group 50.0 months after transplant as compared to 32.8 months in the *de novo* MN group. Four of eleven patients (36%) in the recurrent MN group were female, while seven of the fifteen patients (47%) in the *de novo* MN group were female. The most common race/ethnicity in both the recurrent and *de novo* MN cohorts were white, non-Hispanic or Latino patients. None of the patients in the recurrent MN group had a history of Hepatitis B or C positivity, whereas two of the fifteen patients in the *de novo* MN group had a history of Hepatitis B or C. The rates of concurrent AMR were similar between the recurrent group (36%) and the *de novo* group (40%). The average urine protein to creatinine ratio in the recurrent MN cohort was 2.43 g/g as compared to 1.48 g/g in the *de novo* MN cohort, but this difference was not statistically significant. Serum creatinine levels at the time of MN diagnosis were comparable between the groups, with an average of 1.88 mg/dL in the recurrent MN group and 1.40 mg/dL in the *de novo* MN group. Likewise, the rates of allograft failure were also similar, with a failure rate of 55% in the recurrent MN group and 53% in the *de novo* MN group.

**Table 1:** Patient demographics and clinical features.

	Recurrent MN (n=11)	De Novo MN (n=15)	p-value^a^
Age at time of biopsy in years, average (range)	51.7 (25-70)	44.0 (6-74)	0.32
Time post-transplant to MN^b^ diagnosis in months, average (range)	50.0 (6-142)	32.8 (2-133)	0.87
Female	4 (36%)	7 (47%)	0.60
Self-reported Race/Ethnicity			
White, not Hispanic or Latino	5 (45%)	7 (47%)	0.45^f^
White, Hispanic or Latino	2 (18%)	4 (27%)	
Middle Eastern	2 (18%)	0 (0%)	
Asian	2 (18%)	3 (20%)	
Black or African American	0 (0%)	1 (7%)	
Hepatitis B or C Positive	0 (0%)	2 (13%)	0.20
Concurrent Antibody-Mediated Rejection	4 (36%)	6 (40%)	0.85
UPCR^c^ at diagnosis (g/g), average (range)	2.43 (0.12-10.03)	1.48 (0.10-4.17)	0.40
Serum Cr^d^ at diagnosis (mg/dL), average (range)	1.88 (0.87 – 3.73)	1.40 (0.5 – 2.1)	0.14
Allograft failure^e^	6 (55%)	8 (57%)^g^	0.95

^a^p-value as compared between the recurrent disease category and de novo disease category. Significance was defined as a p-value <0.05.

^b^MN, membranous nephropathy.

^c^UPCR, Urine Protein to Creatinine Ratio.

^d^Cr, Creatinine.

^e^Allograft failure defined as return to dialysis or re-transplant.

^f^Pearson’s chi-square test of independence p-value for the distributions of patient race/ethnicity.

^g^One patient in the de novo group died due to causes unrelated to renal function and was censored from this analysis.

Next, we compared the biopsy findings in the recurrent and *de novo* MN groups (**Table 2**). Positive PLA2R staining by IF was seen more frequently in recurrent MN (64%) compared to *de novo* MN (33%), but this difference was not statistically significant. Somewhat unexpectedly, the mean IgG intensity observed by IF in glomerular capillary loop immune complex deposits was significantly higher in the recurrent group (2.7+ on average) compared to the *de novo* group (1.7+ on average; p-value of 0.002; **Table 2**). No significant differences were observed in IF intensities of C4d or C3 stains. Kappa and lambda IF stain results were available in 5 of 11 cases of recurrent MN and 8 of 15 cases of *de novo* MN, and none of the cases revealed convincing evidence of light chain restriction. Markers of chronicity, including the number of globally sclerotic glomeruli, and the extent of interstitial fibrosis (Banff ci score) and tubular atrophy (ct), were not significantly different between the two groups (**Table 2**). Similarly, we found no significant differences in Banff histologic markers of rejection, including glomerulitis (g), tubulitis (t), peritubular capillaritis (ptc), or intimal arteritis (v) in the recurrent MN and the *de novo* MN cohorts.

**Table 2:** Biopsy features.

	Recurrent MN (n=11)	De Novo MN (n=15)	p-value^a^
PLA2R^b^ positivity by IF^c^	7 (64%)	5 (33%)	0.13
Mean C4d intensity by IF^c^ (0-3/3)	2.5+	2.1+	0.12
Mean C3 intensity by IF^c^ (0-3/3)	1.2+	1.4+	0.62
Mean IgG intensity by IF^c^ (0-3/3)	2.7+	1.7+	0.002*
Percent global glomerulosclerosis, average	9%	20%	0.14
Banff histologic scores			
g (glomerulitis)** **	0.8	0.8	0.93
cg (GBM double contours)** **	0.3	0.1	0.66
ptc (capillaritis)** **	1	0.4	0.14
C4d (C4d deposition)** **	0.6	0.6	0.89
i (interstitial inflammation)** **	0.5	0.6	0.94
t (tubulitis)** **	0.8	0.7	0.75
v (intimal arteritis)** **	0	0.2	0.39
ci (interstitial fibrosis)** **	1	1.2	0.62
ct (tubular atrophy)** **	1.1	1.3	0.62

^a^p-value as compared between the recurrent disease category and de novo disease category.

^b^PLA2R, Phospholipase A2 Receptor.

^c^IF, Immunofluorescence.

*Significance was defined as a p-value <0.05.

**Figure 2:**
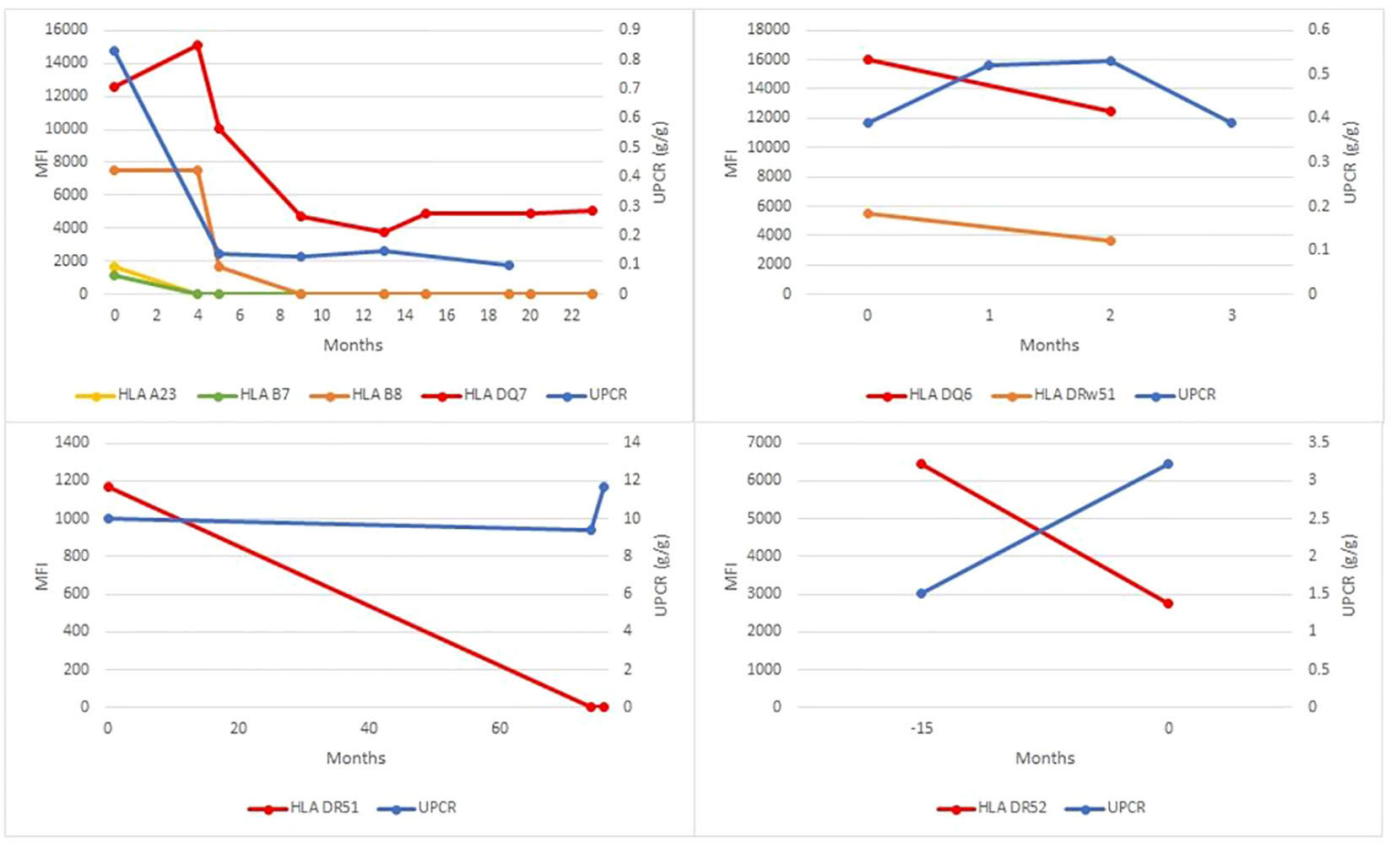
dnDSA titers and proteinuria levels in cases of recurrent MN with concurrent antibody mediated rejection. Four patients with recurrent MN and concurrent AMR were identified. The mean fluorescence index (MFI) of each dnDSA is recorded over time in months relative to MN diagnosis (time point 0). MFI trends of all detected HLA class I and II antibody specificities are shown (left y-axis); the most persistent dnDSA are presented as red lines, the second most persistent dnDSA are presented as orange lines (when present), and any other dnDSA are presented with lines of other colors (when present). Concurrent spot urine protein-to-creatinine ratios (UPCR) trends are shown as blue lines (right y-axis).

**Figure 3:**
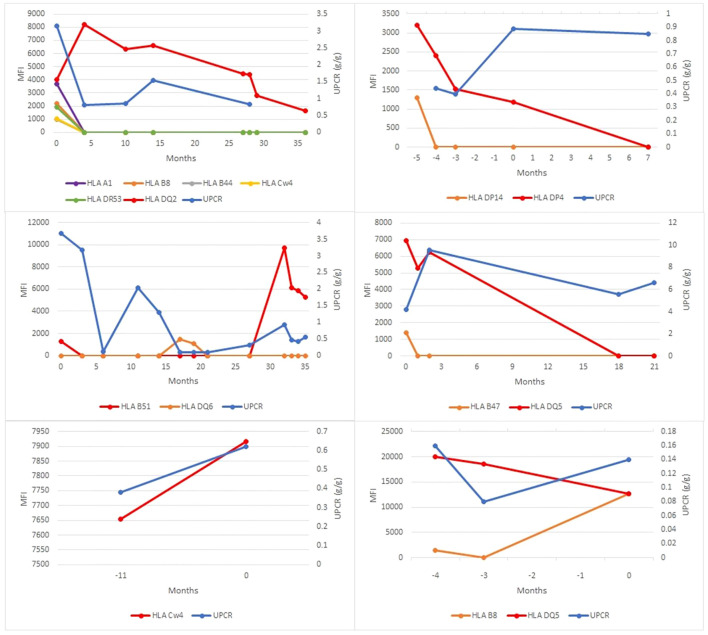
dnDSA titers and proteinuria levels in cases of *de novo* membranous nephropathy with concurrent antibody mediated rejection. Six patients with *de novo* MN and concurrent AMR were identified. The mean fluorescence index (MFI) of each dnDSA is recorded over time in months relative to MN diagnosis (time point 0). MFI trends of all detected HLA class I and II antibody specificities are shown (left y-axis); the most persistent dnDSA are presented as red lines, the second most persistent dnDSA are presented as orange lines (when present), and any other dnDSA are presented with lines of other colors (when present). Concurrent spot urine protein-to-creatinine ratios (UPCR) trends are shown as blue lines (right y-axis).

We further focused on the 10 identified patients with post-transplant MN who also had AMR (**Table 3**), including 6 patients with recurrent and 4 patients with *de novo* MN. While the number of patients in the two groups was too small for robust statistical comparisons, we did not observe obvious differences between recurrent and *de novo* cases with AMR. Similar to the entire cohort of post-transplant MN (**Table 1**), the subset with AMR showed a similar average time to MN diagnosis in the recurrent and *de novo* groups. None of the patients displayed preformed DSA (those detected prior to transplant). In both groups, at least half of the patients developed dnDSA concurrent with MN diagnosis (75% in recurrent MN vs 50% in *de novo* MN), while the rest showed the emergence of dnDSA that preceded MN diagnosis (by 4 to 11 months in three *de novo* MN patients and by 15 months in a single recurrent MN patient). Furthermore, no statistically significant differences were observed in the frequency of single vs multiple DSA seropositivity, PLA2R stain positivity, or rates of allograft failure between recurrent and *de novo* MN subgroups with AMR (**Table 3**). Similarly, a time series analysis of DSA titers and proteinuria levels relative to the time at which MN was diagnosed did not show a uniform correlation between the two or convincing differences between the recurrent and *de novo* MN subgroups (**Figures 2**, **3**).

**Table 3:** Patients with post-transplant MN and AMR.

	Recurrent MN (n=4)	De Novo MN (n=6)
Months post-transplant at diagnosis of MN^a^, average and (range)	64 (33-79)	55 (3-132)
Patients with dnDSA^b^ preceding MN diagnosis	1 (25%)	3 (50%)
Patients with dnDSA concurrent with MN diagnosis	3 (75%)	3 (50%)
Patients with PLA2R^c^ IF^d^ positivity	3 (75%)	3 (50%)
Patients positive for multiple dnDSA specificities	2 (50%)	5 (83%)
Patients positive for a single dnDSA specificities	2 (50%)	1 (17%)
Patients with allograft failure	3 (75%)	3 (60%)^e^

^a^MN, Membranous nephropathy.

^b^dnDSA, de novo donor-specific antibodies.

^c^PLA2R, Phospholipase A2 Receptor.

^d^IF, Immunofluorescence.

^e^One patient in the de novo MN group died due to causes unrelated to renal function and was censored from this analysis.

## 4 Discussion

This case series of post-transplant MN from a single institution is among the largest reported to date (15, 17, 21). Comparison of clinical and biopsy findings in 11 cases of recurrent MN and 15 cases of *de novo* MN did not reveal significant differences in demographic characteristics, time interval after transplant to the diagnosis of MN, laboratory values of renal function and proteinuria, frequency of concurrent AMR, or rates of allograft failure. Aside from a higher average IgG intensity of immune complex deposits observed by IF in recurrent MN compared to *de novo* MN (2.7+ vs 1.7+, p=0.002), no other significant histologic differences were identified in the biopsies at the time of MN diagnosis. The observed apparent difference in IgG intensity is unlikely to be a useful marker at the level of individual biopsies, but the apparent difference overall may indicate a trend for larger and/or more frequent immune complex deposits in recurrent MN as compared to *de novo* MN at the time of diagnosis. Of note, no apparent corresponding difference was observed in C3 intensity, which has been proposed as a possible marker of disease activity in lupus MN (22).

Similar to prior reports, we observed a higher proportion of PLA2R-positive cases in recurrent MN (64%) compared to *de novo* MN (33%), but the difference between the two groups was not statistically significant. Our results suggest a higher incidence of PLA2R-positivity in *de novo* MN than previously reported (6-8%) (15, 21) and question a prior proposals that PLA2R staining is highly specific for recurrent disease (15). In our cohort, the estimated sensitivity and specificity of PLA2R stain for recurrent MN are 64% and 71%, respectively. These findings are further supported by other reports highlighting PLA2R-positive cases in *de novo* MN (16, 23). We note that this apparent discrepancy may be explained, at least in part, by the less stringent definition of PLA2R positivity used in this study. We did not require a PLA2R-positive stain during the initial biopsy diagnostic of MN and accepted a positive stain in any subsequent follow-up biopsy when available (**Figure 1**).

We further focused on the question of a possible link between *de novo* MN and AMR. In addition to the similar rates of concurrent AMR seen in the recurrent MN (36%) and *de novo* MN (40%), the subset of cases with AMR did not reveal significant differences in rates of PLA2R positivity, Banff histologic markers of AMR (g, ptc, C4d), the timing of dnDSA emergence relative to MN diagnosis, positivity for single or multiple dnDSA specificities, or rates of allograft failure. Furthermore, no consistent correlation was seen between proteinuria levels and dnDSA titers in either recurrent or *de novo* MN cases. These results do not provide support for an association between AMR and *de novo* MN. However, the observed rates of AMR in both recurrent and *de novo* MN subgroups observed in this study (36-40%) are higher than that seen in allograft biopsies in general (1-21%) as previously reported (24). Therefore, the possibility that there may be a mechanistic link between MN and AMR, independent of PLA2R status or whether MN is recurrent or *de novo*, remains an open question. This possibility is particularly intriguing in light of the emerging view that MN is an autoimmune disease driven by autoantibodies to various cellular antigens (3–5), which has a compelling mechanistic overlap with the pathophysiology of AMR mediated by DSA that target HLA and non-HLA antigens.

In our series, allograft failure rates were similarly high in recurrent MN (55%) and *de novo* MN (57%). However, we did not find evidence of worse allograft survival in *de novo* MN compared to recurrent MN reported in a prior large multicenter study of post-transplant MN (21). Importantly, MN cases with concurrent AMR had even higher rates of allograft failure (75% in recurrent MN and 60% in *de novo* MN). However, none of the differences were statistically significant due to the limited number of cases in each subgroup. Despite this limitation, our findings highlight the importance of DSA monitoring in cases of post-transplant MN for possible early detection of concurrent AMR.

## Data Availability

The raw data supporting the conclusions of this article will be made available by the authors, without undue reservation.
